# Neuronal Autophagy by the Numbers

**DOI:** 10.1080/27694127.2022.2163091

**Published:** 2023-01-11

**Authors:** Sydney E. Cason, Saurabh S. Mogre, Elena F. Koslover, Erika L. F. Holzbaur

**Affiliations:** a Department of Physiology, University of Pennsylvania, Philadelphia, PA, USA; b Department of Physics, University of California San Diego, La Jolla, CA, USA

**Keywords:** autophagosome, axon, computational modeling, lysosome, neuronal autophagy

Neurons are highly dependent on macroautophagy/autophagy to maintain cellular homeostasis across lifetimes ranging 90 years or more in humans. These cells have high metabolic demands, and are also highly polarized, with axons that can extend up to a meter. Together, these features impart considerable stress, highlighting the importance of stress-relieving pathways such as autophagy. Genetic studies highlight the importance of autophagy, as knockout or mutation of associated genes is sufficient to induce neurodegeneration; defective autophagy is implicated in neurodegenerative diseases including Parkinson and amyotrophic lateral sclerosis (ALS).

Accumulating evidence indicates that autophagy is differentially regulated in neurons as compared to other cell types. In non-neuronal cells, autophagy is generally activated as a stress response. In neurons, however, there is a robust pathway for basal autophagy to maintain cellular health. The overall dynamics of this pathway are well understood: autophagosomes are generated at pre-synaptic sites and the axon terminal. Once generated, autophagosomes fuse with late endosomes or lysosomes (collectively, endolysosomes), and gradually mature to become degradatively competent organelles. These organelles, collectively known as autophagic vesicles (AVs), are recruited to axonal microtubules via associated molecular motors. After an initial period of bidirectional motility along the microtubule, AVs transition to highly processive, unidirectional motility toward the soma. This motility is driven by the microtubule minus-end-directed dynein motor, in concert with DCTN (dynactin) and activating adaptors. Importantly, as AVs are translocated toward the soma, they continue to mature; maturation facilitates the breakdown of internalized cargo and the reuse of constituent macromolecules within the soma.

To develop a quantitative understanding of axonal autophagy, we pursued a collaborative project [1] combining neuronal imaging and computational modeling. This dual approach allowed for a quantitative exploration of the interplay between organelle transport and fusion. Additionally, our work evaluates the effects of axonal geometry in determining spatiotemporal organization. We collected data from primary hippocampal neurons, but our findings are likely to be generally applicable as the pathway is conserved from *C. elegans* to human neurons.

We constructed a general mathematical modeling framework for describing transport and interaction of organelles in narrow cellular projections, represented as advection-reaction processes on concentration fields of different organelle states. The model successfully described key features of the pathway, and highlighted several under-appreciated features of organelle dynamics, including the importance of domain branching and fusion between counter-current flows of particles in determining spatial organization. By matching parameters of the model (production rates, fusion probability) with experimentally-derived observations, we gained insight into features of the maturation process that were not themselves directly observable. The resulting computationally derived and experimentally tested model provides quantitative insights into each step in the pathway of autophagosome biogenesis, motility, and maturation along the axon.

**Figure 1.**
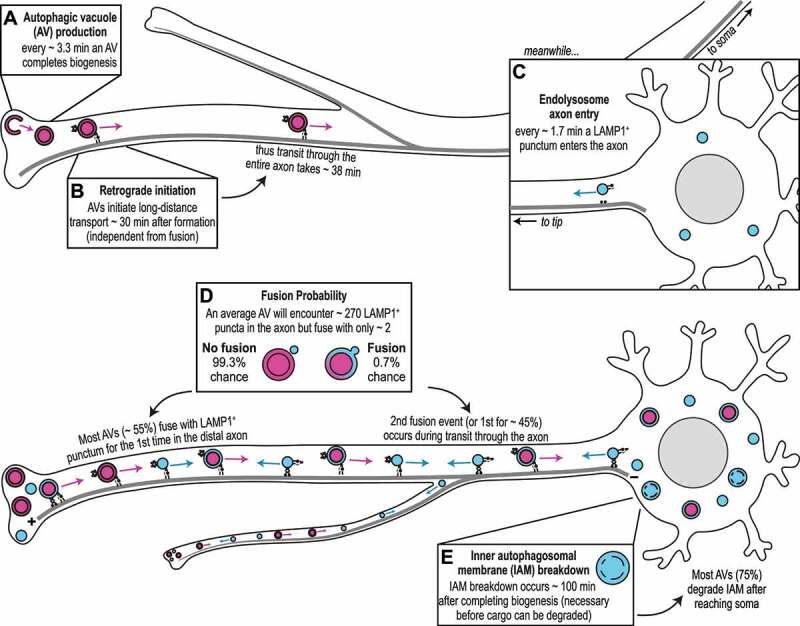
Integrated model for autophagic maturation in the axon, based on quantitative image analysis and computational modeling. Here, we illustrate major findings including the timing of autophagosome biogenesis (**A**) and autophagic vesicle (AV) transport (**B**), lysosome entry into the axon (**C**), and the dynamics underlying AV maturation (**D-E**).

As illustrated in Figure 1, we find that autophagosomes are generated in the distal axon of hippocampal neurons at a rate of 3.3 min^−1^. Most (~55%) of these distal autophagosomes will become LAMP1-positive by fusing with an endolysosome while still in the distal axon. Microtubule binding and the initiation of retrograde transport of the resulting AVs occurs within 30 min of biogenesis and does not depend upon a prior fusion event. AVs are transported along the axon at rates of 0.05 mm/min, arriving at the soma within 40 min on average in hippocampal cultures 7-10 days in vitro, with axons projecting about 1 mm. Of note, we found that a model with a single axonal projection per neuron was unable to fully explain experimental observations, and that incorporating axonal branching was necessary to match observed ratios of endolysosomes to AVs of 1.3:1 in the distal axon and 2:1 in the proximal axon.

Two very surprising findings resulted from our work. First, the high density of endolysosomes along the axon ensures that each newly generated autophagosome has the potential to encounter ~270 endolysosomal organelles during the journey from distal to proximal axon. However, our modeling predicts an average of only about 2 fusions per AV, indicating a very low fusion probability, on the order of 0.7% per encounter. Thus, fusion appears to be a tightly regulated step.

**Figure 2.**
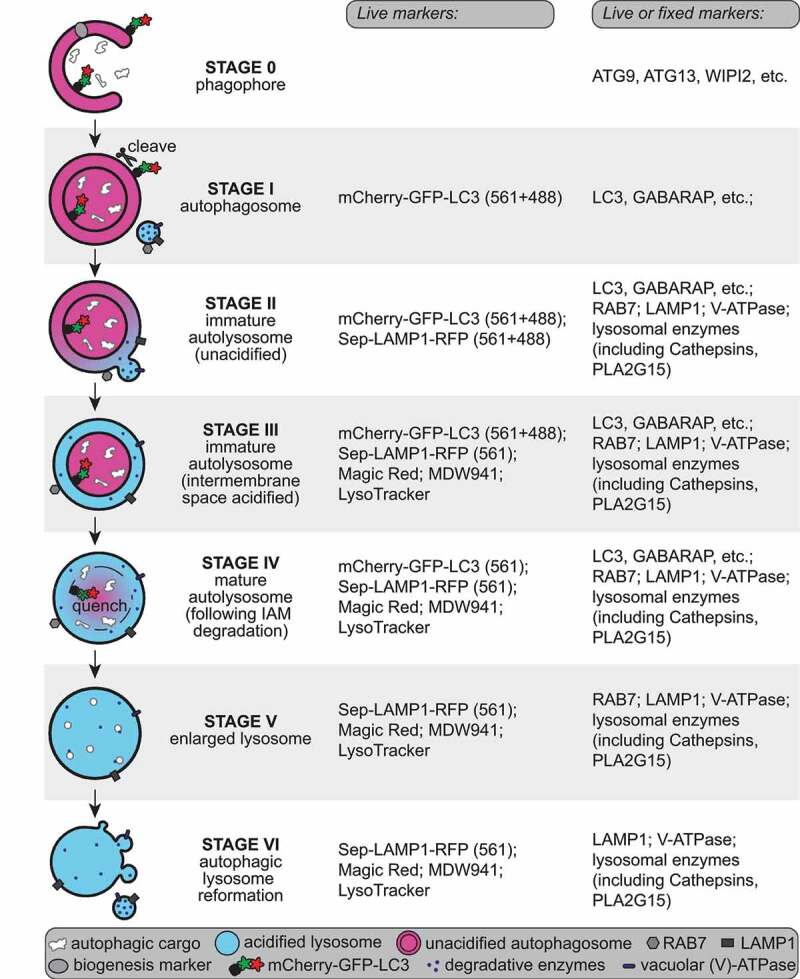
Distinct stages of autophagosome biogenesis and AV maturation in neuronal axons. The stages of autophagosome biogenesis and AV maturation can be distinguished using the indicated tools for the analysis of live or fixed neurons. These approaches highlight the striking delay observed between initial autophagosome-lysosome fusion and inner autophagosomal membrane degradation in neurons, as compared to other cell types. We now define two distinct stages, referred to as immature autolysosomes and mature autolysosomes.

The second surprise came from comparison of markers for the acidification of AVs. Probing for acidification with the dye LysoTracker Red suggested that a large fraction of axonal AVs are acidified. However, experiments with a tandem mCherry-EGFP-LC3 reporter – in which the EGFP but not the mCherry moiety is pH-sensitive – revealed a different result. We found that the core central lumen of most axonal autophagosomes, where the tandem reporter localizes, is not acidified. The apparent discrepancy is because LysoTracker reports on the acidification of the inter-membrane space between the outer and inner autophagosomal membranes, following initial fusion of the autophagosome with an acidified endolysosome. However, full maturation and degradation of engulfed cargo requires breakdown of the inner autophagosome membrane (IAM) followed by acidification of the lumen, which is measured by the tandem mCherry-EGFP-LC3 reporter (Figure 2). In mouse embryonic fibroblasts, these steps are separated by only about 6 min, but in neurons we found that IAM degradation, and thus full maturation to a degradation-competent organelle, occurs about 100 min after biogenesis. Of note, this delayed maturation is not due to endolysosomal immaturity in the axon, because the majority (~80%) of axonal endolysosomes colocalize with both the vacuolar ATPase and degradative enzymes, including the phospholipase PLA2G15, the homolog of which was recently identified in *C. elegans* as the lipase responsible for IAM degradation.

Thus, the majority of autophagosomes reaching the soma have fused with 1-2 endolysosomes but are not yet fully mature degradative organelles. Why might maturation be so slow in neurons? Here, we can only speculate, but recent work from our group has shown that mitochondrial DNA (mtDNA) is enriched within the lumen of axonal autophagosomes. The tight regulation of autophagosome maturation may prevent the local release of mtDNA, a known danger/damage-associated molecular pattern/DAMP along the axon. Neuronal axons are relatively vulnerable, with axonal die-back a common early marker of pathology in neurodegeneration. Thus, we propose that axonal autophagy acts as a homeostatic mechanism that is carefully tuned to maintain axon health to ensure neuronal survival over the decades, while defects in this pathway may enhance vulnerability to neurodegenerative disease.
